# The Politicization of Research Ethics and Integrity and its Implications for Research Governance

**DOI:** 10.1007/s10805-024-09563-2

**Published:** 2024-10-28

**Authors:** Ian Slesinger, Kadri Simm

**Affiliations:** 1 Ethics, Human Rights and Emerging Technology Research Cluster, Trilateral Research Ltd., London, UK; 2 Department of Philosophy, University of Tartu, Tartu, Estonia

**Keywords:** Research ethics, Ressearch integrity, Research misconduct, Politicization, Research governance, Case studies

Whilst research has always been political and politicized, an emerging theme in the area of research ethics and integrity (REI) is the increased politicization of REI itself in areas of scientific and/or political controversy such as climate change, gender dysphoria treatment, the management of pandemics and women’s reproductive rights. One aspect of this trend is the ‘weaponization’ of research misconduct allegations and findings to discredit individuals, institutions or intellectual or scientific positions (Bruckner, 2024). Such politicization has been amplified by the increasing role of online and social media as a platform for public discourses on REI, and for airing whistle-blower complaints and accusations of Research Misconduct (RM) against both research-performing institutions and individual researchers. This shift has implications for the temporality of REI reporting and investigations, since traditionally such processes are slow and deliberative to ensure transparency, objectivity, fairness and thoroughness. However, this meticulous approach is inimical to the fast pace of online media discourse (including social media), which plays an increasing role in RM cases and adds pressure on institutions and researchers to accelerate the speed of judgments in RM cases and to make their investigations and findings more publicly transparent.

This piece will discuss three cases that highlight multiple aspects of the politicization of REI, which were identified through a qualitative case study exercise carried out by the Horizon Europe-funded BEYOND project (https://beyondbadapples.eu) to assess novel and emerging issues related to REI discourses. The study conducted a comparative qualitative evaluation of press and public discourses around 23 identified real-life cases of (alleged) research misconduct that occurred between 2019 and 2023 across four project partner countries: Estonia, Latvia, the Netherlands and the UK (Slesinger et al., 2023). Cases were sourced from multiple online and social media outlets in English and local languages,^1^ which were then cross-referenced with independent sources including investigatory reports, institutional statements, Retraction Watch, and professional online profiles. This method enabled the extraction of rich qualitative data with detailed narratives and factual information on each case, addressing the challenge of limited databases for RM reporting in Europe. While the limited number of countries involved and the methodology employed prevent broad conclusions, key issues were identified for initial discussion and further enquiry.

Scientific considerations have often been political in terms of truth claims, funding, acceptable topics or approaches to research, and more recently, in terms of research ethics regulations (Chelli & Cunliffe, 2022; Jaschik, 2007; Schmid-Petri et al., 2022; Solbakk et al., 2021). Politization can involve attempts at delegitimization (Briggle, 2009), contestation (Palonen, 2003) and reducing a research position to “corruption, hypocrisy, and narrow self-interest” (Brown, 2009). Of course, not all politicization of research is inherently problematic, especially if research is viewed as a taxpayer-funded social practice that contributes towards socially valued goals (so-called politics with large “P” (Pellegrino, 2006). What is novel here though is that the traditionally bureaucratic and expert-focussed domain of REI, and claims about RM, are shifting from the remit and concerns of institutional governance into the public sphere as part of the broader context of contemporary political polarization. This has implications for the nature and temporality of REI investigation, remediation, and mitigation processes. It introduces new scrutiny and an urgency for swift action into a traditionally technocratic domain of adjudication and mediation, which typically operates at a slower pace to facilitate methodical and careful evaluation of complex evidence. This dynamic must also be understood in relation to the socio-political contexts of the places in which these shifts are taking place. This relates to the different cultural orientations towards trust in science and the rise of populist political movements in both certain European countries and globally, which situate (mis)trust of scientific expertise within a broader anti-elite epistemology and rhetoric.

The politicization of REI has multiple dimensions and manifests differently in various contexts. This includes the contexts of how expertise confers authority in political controversies in areas including: gender, environmental concerns, the character of the nation-state, individuals’ rights to privacy, and contestations over the relationship between researchers’ positionalities and the production of knowledge. Below we discuss three cases in more detail to flesh out some of those dimensions.

In August 2023 thousands of young Estonian women received an e-mail invitation to participate in the population survey on childlessness. The survey asked highly sensitive questions about sexual lifestyle, partnerships and reasons for not having children. The survey questions initially sparked concerns due to their perceived biased wording and an unorthodox mix of topics (for example both political and sexual preferences), a concern likely shaped by the ideological reputation of the organization overseeing the survey (Pärli, 2023). The study was officially commissioned by Pere Sihtkapital, a population policy think tank established and funded through the initiative of the national conservative party Isamaa (Fatherland) (Eylandt, 2018). As the think tank did not have the right to access the contacts of childless Estonian young women through the Population Register, they partnered with University of Tartu which does have this right through their research accreditation. In this case there was a convenient link – a Tartu professor was both a member of the think-tank’s council and a dean of the university faculty and signed the contract on behalf of the university (a contract that the university now claims is fictional and void, as it was signed both against the advice of the university lawyer and in violation of anti-corruption regulations)(Asser, 2023). Initial concerns around the survey in the media were published on a Friday, the media storm continued throughout the weekend and on Monday the university decided to end the employment contract of the prominent professor. Three reasons were cited by the university, amongst them not complying with the requirements of research ethics and integrity (Asser, 2023).

In this instance, the insensitive mixing of sexual and political subjects and the political nature of the organization undertaking the study created a media maelstrom where REI issues became central. The politicization of research was decried both by those critical of the survey and those who supported it. And while research ethics infringements really were significant here, concerns have later been raised about the possibility of “employing the code of ethics as a whip” (Soomere, 2023). Furthermore, the intense media attention this scandal received and the rapid pace at which it escalated forced the university to react quickly to address the allegations and control the resulting public relations damage. While the long-term impact of the scandal on Estonians’ willingness to participate in research remains to be seen, in the short term, thousands rushed to the population registry to opt out of any future research involvement in the wake of the scandal (Liive, 2023).

Another case from the UK highlights the complexity of the milieu in which the politicization of REI concerns occurs and how this politicization has implications for both researchers and society. A 2019 investigation by the BBC’s NewsNight current affairs programme found that a prominent UK gender dysphoria clinic lowered the age at which it would provide children puberty blockers based on information from a study started in 2011 at the clinic that had since come under scrutiny (Cohen & Barnes, 2019). Critics argued that the study had significant flaws both in terms of research integrity (RI) and research ethics (RE). The absence of a randomized control group meant that it was impossible for researchers to tell whether there was a causal relationship between some participants’ reports of thoughts of self-harm and suicide and the hormone-blockers being trialled. The study authors claimed there was no meaningful correlation due to the small sample size (Cohen & Barnes, 2019), and there were ethical and clinical justifications for not using a randomized control group (Health Research Authority, 2019). Ethical concerns were also raised about child participants’ autonomy to consent, long-term health risks and participants’ degree of informed consent to choose alternative and later approaches to medical transitioning. It also led to a series of legal interventions in the UK’s High Court around the capacity of under 16s to consent to such treatment. However, the UK Health Research Authority (UKHRA) cleared the initial study of wrongdoing in an investigation carried out in response to concerns raised in the NewsNight episode (Health Research Authority, 2019). The actual clinical decision to treat under 16s was itself not research misconduct or a breach of research ethics according to the UKHRA report, as ethics in clinical practice is distinct from RE and beyond the UKHRA’s remit, although the clinic was critiqued in the report for not adequately differentiating, both internally and externally, the distinction between research activities and clinical practice in internal discussions and in communication with external stakeholders and the public. However, concerns that the study was used as an underlying justification for the change in clinical practice subsequently became the centre of controversy. Furthermore, the controversy over the clinical treatment of under 16s for gender dysphoria has resonances with wider political debates around transgender rights. This case also highlights how the politicization of REI issues can have a chilling effect on researchers. In relation to this controversy, some researchers and clinicians stated that they became reticent to express their views on adolescent gender dysphoria or question research findings and methodologies in the field due to fear of being accused of transphobia (Brooks, 2023).

In other instances RM allegations have become politically instrumentalized or ‘weaponized’ in order to discredit a position or particular researcher in an area of controversy. This is highlighted in a 2023 case from the UK, which concerned Low-Traffic Neighbourhoods (LTNs). LTNs is a practice adopted by the Greater London Authority to encourage walking and cycling by blocking off side streets to motor vehicle traffic in residential areas, which has faced dissensus from some motorists and became embroiled in wider partisan political conflicts between the UK’s then Conservative central government and the Labour-led Mayor of London’s office (BBC News, 2023). In this case, a video was released to a local residents’ WhatsApp group (and subsequently the press) by a shopkeeper showing a mid-career researcher who advocated for Low-Traffic Neighbourhoods (LTNs) tearing down an anti-LTN petition poster in a local shop. As a result the researcher’s academic independence was called into question, and she was accused of research misconduct by committing actions that undermined her position as an ‘independent’ academic expert(Parry, 2023; Russell, 2023). Her institution subsequently cleared her of committing RM and decided to take no further action following a review carried out by the institution’s Research Governance and Integrity Office to decide whether a full investigation would be necessary (Bird, 2023). The institution also affirmed her entitlement as a “’published expert in the impact of LTNs’… to give evidence in legal proceedings.” It is likely that the researcher’s personal conduct was not conducive to democratic deliberation, and possibly unprofessional considering her position as a researcher on a topic of controversy. However, these points are separate from the issue of research misconduct based on accusations of bias, which sought to undermine the credibility of both the researcher and her underlying position in favour of LTNs. This required the institution to navigate complex issues around how researcher’s positionality influences research, how positionality should be addressed and questions about the extent to which advocacy is perceived as problematic in academic contexts. This case underscores the gap between academic discourse, which delves into nuanced discussions around researcher positionality, and the public perception of scientists as unbiased or “neutral”.

Two factors are noteworthy here in light of these cases of politicization of REI. One is the political ‘weaponization’ of REI claims and allegations to discredit individuals, institutions or ideas and scientific concepts with which the group or individual making accusations of research misconduct disagrees (Bruckner, 2024). This tactic uses purported REI concerns as a means to undermine the credibility of an individual or institution, or the epistemological foundations of a concept that is being contested. The other factor is the role of online and social media as a medium and catalyst for the agitated and amplified content and tone of public discourse. Whilst our research does not point to social media as the main causal factor behind the increasing politicization of REI concerns, it does play a significant role in how REI concerns are identified, addressed and navigated both within institutions and in wider public discourse. As a medium, online and social media can both encourage the simplification of complex issues for ease of communication and clear messaging, and amplify the speed and tenor of how public controversies are exposed and played out. It can be hypothesized that there is some form of interplay between the polarized and amplified nature of social media discourse and the ‘weaponization’ of REI concerns. However, the current extent and modalities of this potential nexus are unclear and merit further examination. Yet, the increased public discourse on REI allegations can also be seen as beneficial. Online platforms and social media have facilitated whistleblowing and provided avenues for less empowered individuals, both within academia and beyond, to voice their concerns. These altered circumstances around REI allegations and investigations must also prompt institutions to reconsider their approaches to handling such matters.

Given that politicized REI accusations move fast and are capable of creating significant individual and institutional damage, institutions, REI experts and individual researchers must be prepared for the risk of ethical standards being instrumentalized for other purposes. This requires institutions and individual researchers to be strategically proactive in implementing counter-measures to prevent the misuse of REI concerns. We conclude with some recommendations towards this:

- More comprehensive and in-depth qualitative monitoring of case studies of alleged REI to empower REI authorities, institutions and individual researchers to analyse patterns and trends in the politicization of REI concerns and the political weaponization of REI processes. This knowledge can be applied to develop robust evidence-led approaches to handling REI politicization and weaponization (Slesinger, Ian and van den Hooff, Susanne, 2024).
- Institutions and researchers should be clear in communicating that REI issues are distinct from the sensitive or politicized nature of the research itself, and be clear in defining the scope of REI. This includes transparency in acknowledging the inherently social and political dimensions of research, thereby rejecting unrealistic expectations of political neutrality.
- In response to allegations, institutions should prioritize swift acknowledgment of raised concerns, emphasizing transparency in the investigative process and providing clear timelines for resolution. This requires institutions to allocate adequate resources to facilitate transparent and expedited investigations and resolutions of REI accusations. By investing in adequate systems, such as training for investigators and streamlined procedures, institutions can both improve the efficiency and transparency of addressing allegations and ensure a commitment to ethical standards.
- Although this piece mostly treated REI as a single phenomenon, it could be worthwhile to consider an analytical distinction between research ethics and research integrity in addressing politicized REI allegations. In other words, how does dishonest or malign research practice differ from shoddy or flawed research practices? This distinction would require that RE and RI be handled differently, with different implications for institutional processes.
- As politization of REI increases the likelihood of false allegations institutions and funding bodies should provide transparent protocols for supporting researchers who have been affected by politically-charged allegations of research misconduct, alongside rehabilitating and reintegrating researchers found to have committed RM. These processes require in parallel the effective and consistent communication of these procedures to all involved stakeholders and the public.
- Individual researchers are public figures due to their academic profiles and usually enjoy a certain level of public trust associated with their affiliation. As such, they must anticipate that their activities and actions outside of their academic work, including personal behaviour and activism, can invite scrutiny of their research. This also can cause their institutional affiliation to be used as a lever to apply pressure on them, or a position they endorse. This requires researchers to engage in reflective practice in relation to sensitive political issues.
- While individual researchers are usually well-aware of the (potentially) contested nature of their research fields, they should address REI concerns in politically-charged research topics explicitly in the risk management of their research projects, and outline proactive mitigation strategies for these risks. This includes care in framing research questions, methodologies and the reporting of findings to prevent the inaccurate framing of research results, and anticipate potential forms of harm to participants as part of the research ethics process.

## Footnotes

1 We will not treat online media and social media as separate domains, since so-called “twitter-storms” are often picked up by online media and vice versa, as social media routinely hashtags and references traditional media reporting.
